# Resection of a ruptured mature cystic teratoma diagnosed two years after the onset of perforation

**DOI:** 10.1186/1477-7819-12-321

**Published:** 2014-10-23

**Authors:** Hiroaki Kuroda, Toshinori Hashidume, Masaoki Shimanouchi, Yukinori Sakao

**Affiliations:** Division of General Thoracic Surgery, National Hospital Organization, Ibarakihigashi National Hospital, 825 Terunuma, Tokai-mura, Naka-gun, Ibaraki, 319-1113 Japan; Division of General Thoracic Surgery, Aichi Cancer Center Hospital, 1-1 Kanokoden, Chikusa-ku, Nagoya, 464-8681 Japan

**Keywords:** perforation of mediastinal teratoma, inhomogeneous, thoracoscopic resection

## Abstract

Perforated cystic teratomas are rare. Our patient was a 16-year-old female who presented with severe chest pain two years ago. A right-sided pleural effusion was accidentally detected by chest radiography performed at her school. Computed tomography revealed a high density area with multiple small, thickened, lobulated lesions and a low density area with pleural effusion adjacent to a thinned wall. Pathognomonic inhomogeneous computed tomography findings led to an accurate diagnosis of a mature teratoma that had ruptured long before presentation. Thoracoscopic resection was performed, and the final histological diagnosis was a mature teratoma with partial rupture.

## Background

Before puberty, approximately 20% of patients with primary mediastinum tumors have germ cell tumors [1]. Germ cell tumors of the mediastinum are believed to be derived from the thymus, and they typically occur with equal frequency in men and women [1]. There have been few published reports of the rate of teratoma rupture; however, a rate of 36.0 to 41.0% has been reported in a high-risk population [2, 3].

We describe a patient who underwent thoracoscopic resection for a mature teratoma that had ruptured two years earlier. A definitive diagnosis was made on the basis of the results of preoperative computed tomography (CT).

## Case presentation

**Figure 1:**
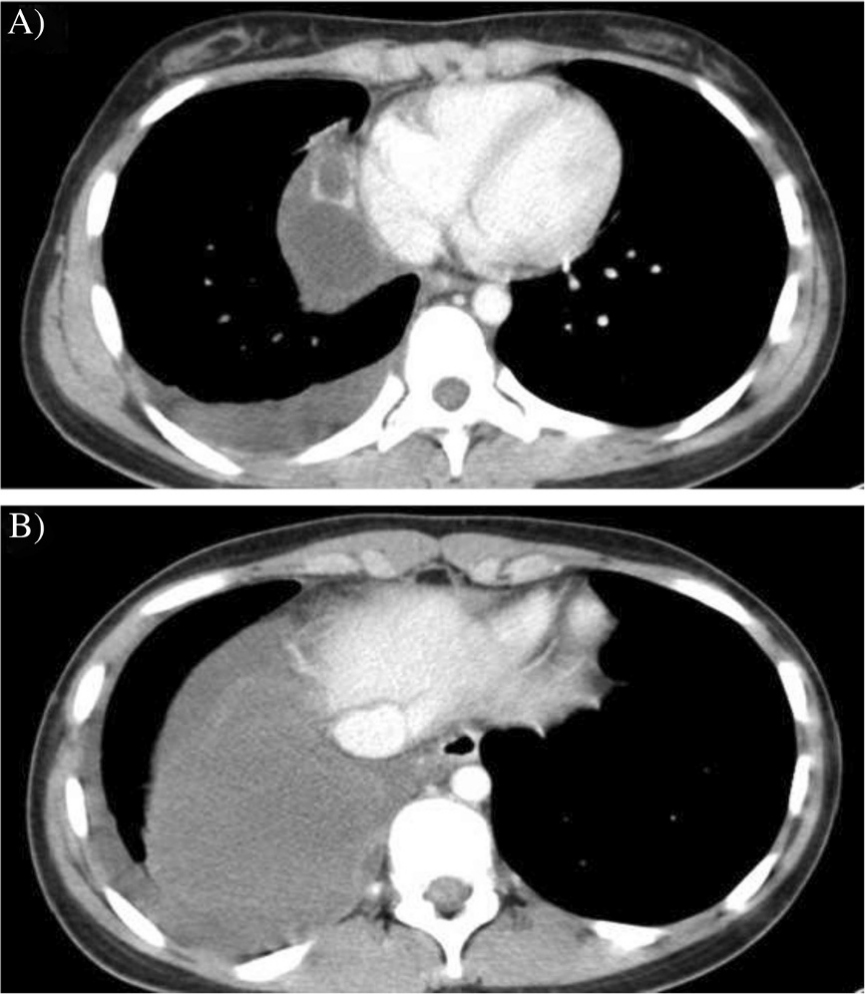
**Computed tomography imaging. (A)** Computed tomography revealed lobulated cystic masses with an enhanced tumor wall in the anterior mediastinam. **(B)** Computed tomography revealed a low density area equal to pleural effusion with an adjacent thin wall.

**Figure 2:**
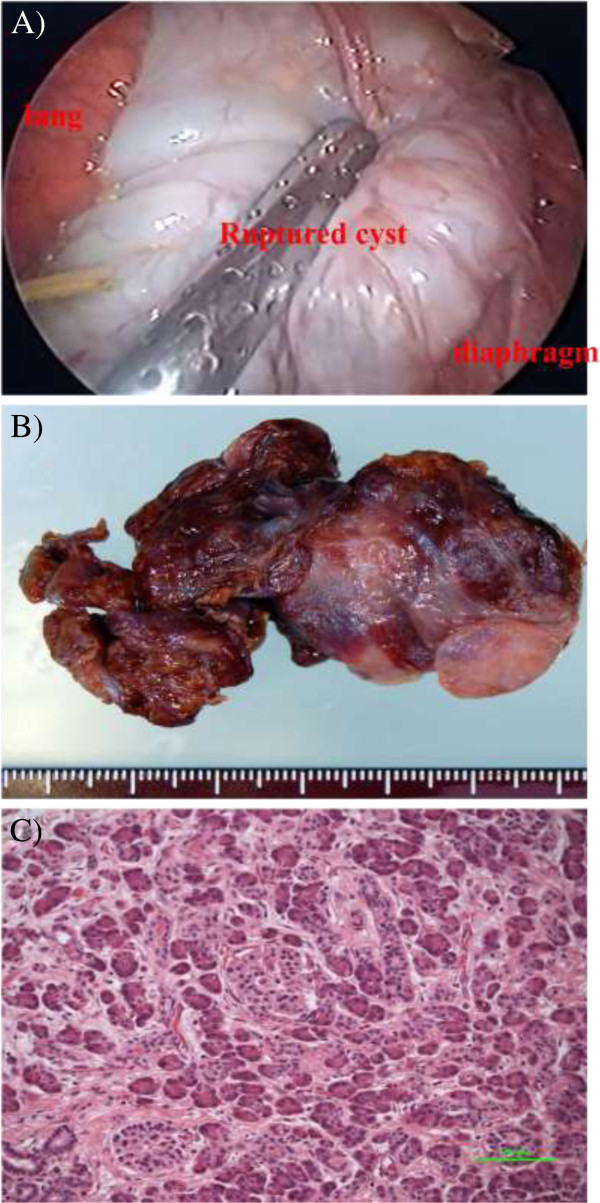
**Thoracoscopic finding, resected specimen and histological features of mature cystic teratoma. (A)** Thoracoscopic image of a smooth-surfaced cyst with a wall between the lower lobe and diaphragm. **(B)** Macroscopic photography showed a ruptured cystic lesion (left) and multiple lobulated cysts (right). **(C)** Histologically, the pancreatic glands and islets of Langerhans were apparent.

A 16-year-old female presented with severe chest pain two years ago. She reported occasional right-sided chest pain and dyspnea on exertion but had never visited a hospital for her condition. Her past history was not significant. Right-sided pleural effusion was identified at an annual health screening conducted at the high school. Contrast-enhanced chest CT (mediastinal window) confirmed two inhomogeneous patterns, that is, a high-density area with multiple, small, thickened, lobulated lesions of a maximum diameter of 25 mm on the anterior mediastinum (Figure 1A) and a low density area with pleural effusion adjacent to a thinned wall on the former caudal side (Figure 1B). On admission, tumor markers (α-fetoprotein, β subunit of human chorionic gonadotropin (hCG), and carcinoembryonic antigen) were within normal ranges. No signs of inflammation were detected by blood examination, and needle thoracocentesis was performed. Infection, secondary effusion due to pneumothorax, and bleeding were all ruled out by analyses of the pleural effusion. Based on the findings of imaging and a systemic examination, a ruptured teratoma was finally suspected.Thoracoscopic resection was performed. A 17 × 10 cm cyst with a smooth surface and thinned wall was found between the lower lobe and the diaphragm (Figure 2A). When we observed the mediastinal side (thymus), small cysts containing various concentrates of lactescent creams were identified (macroscopic findings, Figure 2B). Microscopic examination revealed that the tumor contained squamous epithelium associated with sebaceous glands, aberrant pancreatic tissue containing pancreatic glands, and islets of Langerhans (Figure 2C), and columnar epithelium intermixed with goblet cell and glandular epithelium. Neither immature cells nor malignant transformation was identified.

The patient had no complications during the postoperative period and was discharged on postoperative day 4. At follow-up examination, six months after surgery, she was found to be completely asymptomatic.

### Discussion

Clinical symptoms are always associated with ruptured teratomas, whereas only approximately 50% of unruptured teratomas are symptomatic, and severe symptoms such as chest pain of recent onset or hemoptysis were more frequently observed in patients with ruptured teratomas than in patients with unruptured teratomas [2]. In our case, our patient had severe chest pain two years earlier, but she did not visit the hospital because her symptoms subsided. We conjectured this symptom was the onset of rupture. The tumor was accidentally detected by chest radiography. To the best of our knowledge, there have been few cases in which a ruptured teratoma was diagnosed after a long interval from the time of rupture. Suwatanapongched *et al*. reported an intrapulmonary rupture of mediastinal teratoma after four years [4]. However, we based onset on the diagnosis and only for symptoms in this report, and there is no accurate evidence to confirm this. Our case may have had a later onset than believed.

The following two primary and controversial proposals relating to possible perforation mechanisms have been published: (a) sebaceous materials or digestive enzymes derived from tumor tissue cause inflammation, infection, ischemia, and necrosis [2, 3] and (b) autolysis occurs via digestive enzymes derived from the pancreatic or salivary gland of tumor tissue [2, 5, 6]. The latter hypothesis was most applicable to our case because pathological examination revealed that there was no necrosis or ischemic findings, and the pancreatic duct and islets of Langerhans were outstanding (Figure 2C). Hirawa *et al*. reported that high levels of amylase were noted in pleural fluid that originated from a mediastinal teratoma [5]. The serum amylase level in our patient was within the normal range, and the possibility of dilution with reactive effusion over the preceding two years was considered.

On the basis of their analysis of 17 tumors, Choi *et al*. also reported no significant difference between ruptured and unruptured teratomas in terms of wall thickness, location of the mass, and tumor size [2]. In the present case, the tumor wall of the ruptured cystic lesion (3.8 mm) was thinner than that of an unruptured cystic lesion (6.8 mm). We hypothesized the following mechanisms for this phenomenon: (a) degeneration of the ruptured cystic wall over approximately two years or (b) gradual thinning of the wall due to a rise in pressure.

Approximately 90% of unruptured teratomas present as homogeneous in CT findings [2]. Homogeneity or heterogeneity on CT is one means of distinguishing between ruptured and unruptured teratomas. In addition, Inoue *et al*. reported their short-interval CT finding that an increase in the soft tissue area of the anterior chest wall adjacent to the tumor was extremely helpful for making a definitive diagnosis of teratoma rupture, thereby facilitating prompt treatment by surgical resection [6]. In the present case, the tumor also had a high density area with multiple small thickened lobulated lesions and a low density area with pleural effusion adjacent to a thinned wall (Figure 1A-B); therefore, we could make an accurate diagnosis with these CT findings.

## Conclusions

In conclusion, CT is a useful modality for diagnosing a ruptured teratoma. It is important for surgical resection that a definitive diagnosis is obtained with these CT findings. To the best of our knowledge, there is second report of a ruptured teratoma diagnosed more than two years after the onset of perforation.

## Consent

Written informed consent was obtained from the patient for publication of this Case report and any accompanying images. A copy of the written consent is available for review by the Editor-in-Chief of this journal.

## Abbreviations

CT: computed tomography.
